# Using smartphone accelerometry to assess the relationship between cognitive load and gait dynamics during outdoor walking

**DOI:** 10.1038/s41598-019-39718-w

**Published:** 2019-02-28

**Authors:** Simon Ho, Amelia Mohtadi, Kash Daud, Ute Leonards, Todd C. Handy

**Affiliations:** 1 Department of Psychology, University of British Columbia, Vancouver, British Columbia Canada; 2 School of Psychological Science, University of Bristol, Bristol, United Kingdom

## Abstract

Research has demonstrated that an increase in cognitive load can result in increased gait variability and slower overall walking speed, both of which are indicators of gait instability. The external environment also imposes load on our cognitive systems; however, most gait research has been conducted in a laboratory setting and little work has demonstrated how load imposed by natural environments impact gait dynamics during outdoor walking. Across four experiments, young adults were exposed to varying levels of cognitive load while walking through indoor and outdoor environments. Gait dynamics were concurrently recorded using smartphone-based accelerometry. Results suggest that, during indoor walking, increased cognitive load impacted a range of gait parameters such as step time and step time variability. The impact of environmental load on gait, however, was not as pronounced, with increased load associated only with step time changes during outdoor walking. Overall, the present work shows that cognitive load is related to young adult gait during both indoor and outdoor walking, and importantly, smartphones can be used as gait assessment tools in environments where gait dynamics have traditionally been difficult to measure.

## Introduction

Traditionally, dynamic adjustment of gait is seen as a way to minimize energy cost during ambulation^1,2^, and in fact, humans continuously optimize energetic cost in real-time, even if the savings are small^3^. However, energy conservation is only part of the reason for changing gait dynamics. For example, visual information from the external environment is often used to guide foot placement^4,5^, and in turn environmental complexity changes where we look when walking^6^. The interaction between gait and environment suggests that some level of cognitive processing is required for effective ambulation. Consistent with this, the maintenance of postural and gait control both require attentional resources, and are highly dependent on cognitive function^7,8^. Furthermore, availability of cognitive resources affects our ability to handle environmental challenges (e.g. avoiding obstacles) while walking^9,10^. The external environment also imposes demands on our cognitive systems^11^, and fluctuations in environmental load should be reflected in real-time gait dynamics during outdoor walking. However, gait is difficult to measure in outdoor environments owing to a lack of portable measurement tools, but wearable technology may provide a way to measure gait dynamics in more natural contexts. The present study therefore set out to examine whether the availability of cognitive resources affects gait dynamics during outdoor walking, using a methodology that is more appropriate for real-world gait assessment.

In the laboratory, the relationship between gait dynamics and cognition is often studied with dual-task paradigms: if walking requires cognitive resources, then increasing the difficulty of a concurrent secondary task should result in changes to the dynamics of gait (e.g. speed, step time variability, regularity). This effect has been well documented; for example, research has shown that walking under high cognitive load is associated with worse performance on a stepping stone task^12^, reduced ability to avoid obstacles^10^, reduced gait speed and leg swing time^13^, or increased number of missteps in complex walking environments^14^.

There are a couple of methodological issues in the gait literature that our study aims to address. The first concerns the difference between treadmill and overground walking. Many studies examining the relationship between gait dynamics and cognitive function have used treadmill walking, finding, for example, that variability in gait parameters increases under higher levels of cognitive load^15–17^. However, treadmill walking has been shown to be different from overground walking^18,19^ and can produce significantly reduced variability in stride time and upper and lower body acceleration, even at self-selected speeds^20^. Therefore, findings from treadmill-based studies cannot reliably be generalized to overground walking.

The second methodological issue concerns indoor vs. outdoor walking. Many cognition and gait studies have been conducted indoors due to the use of treadmills or 3D motion capture systems, but these studies don’t usually test the impact of the environment itself on gait, and findings therefore do not necessarily generalize to environments outside of the laboratory. Walking in the lab is likely to be different from outdoor walking as cognitive load can originate from different sources. For example, visual stimuli in urban environments might impose a high demand on cognition, while natural environments may have a restorative effect on attention^11^. In other words, explicitly manipulated load in the lab via a cognitive dual-task may not have the same impact on gait as load that is inherent to an outdoor environment.

The ability to study outdoor gait dynamics would provide an opportunity to assess ambulation in cognitively demanding environments where it has traditionally been difficult to perform gait assessment. For example, crossing the street while under cognitive load has been studied in virtual reality^21^, but gait dynamics are likely to be different at a real intersection where the consequences of unstable gait are more severe. Being able to study environmental effects on gait in the real world could help inform the design of public spaces, such as reducing levels of load at crosswalks to reduce mobility-related traffic accidents.

The competition for cognitive resources between cognition and gait may also contribute to increased fall risk in the elderly (especially in cognitively demanding environments), with divided attention resulting in reduced obstacle avoidance abilities and an increase in missteps^10,14^. Gait dynamics can be used as a proxy for the cognitive demand of an outdoor environment and may help us understand whether certain environments are more cognitively challenging and thus contribute to increased fall risk.

The goal of the present article is to determine whether cognitive load from environmental and task-related sources impacts gait during indoor and outdoor walking, and whether findings from indoor studies generalize to outdoor environments. Findings from previous studies lead to the prediction that gait should become slower and more variable as levels of cognitive load increase, and that this relationship should be observable both indoors and outdoors, regardless of the source of the cognitive load (internal task demands vs. external/environmentally induced). We tested this hypothesis across four experiments. First, we explicitly manipulated levels of cognitive load using a verbal dual-task to determine whether the relationship is detectable using a smartphone during an indoor walk (Experiment 1). Next, we validated the use of fractal dimension from outdoor photos as an operational definition of environmental load (Experiment 2). Finally, we tested whether the load imposed by natural environments, as measured by fractal dimension, impacts gait dynamics during outdoor walking (Experiments 3 and 4).

## Experiment 1

First, we tested the relationship between cognitive load and gait dynamics in a controlled environment as a validation of our smartphone methodology. We predicted that smartphone-based accelerometry, during indoor overground walking, should be sensitive enough to detect a relationship between load and gait, specifically that increased levels of cognitive load would result in slower and more variable gait. To test this, participants were asked to walk down a hallway while vertical acceleration was measured using a smartphone accelerometer. Participants were placed under varying degrees of cognitive load using a verbal dual-task.

### Ethics Statement

Ethical approval was received through the University of British Columbia’s Behavioural Research Ethics Board, written informed consent was obtained from each participant prior to the start of the study, and research was performed in accordance with ethics board guidelines.

### Participants

A power analysis was conducted to determine minimum sample size for the study. We used a conservative effect size estimate of 0.1 (Cohen’s *d*) as we were unsure of the magnitude of the relationship between gait and our chosen cognitive task, especially when measured using smartphone accelerometry. We also expected a violation of the homogeneity of variance assumption owing to differences in step variability across conditions; thus, a non-sphericity correction was applied conservatively at 0.6. The analysis indicated that at least 140 participants would be required to achieve 80% power. A total of 154 participants (mean age = 20.50, *SD* = 3.25, 35 male) were recruited for the experiment. Participants were undergraduate student volunteers and received course credit for their time.

### Apparatus

An LG Nexus 5X smartphone (http://www.lg.com) was used for data collection. This particular model was chosen due to its wide range of integrated sensors; however, any smartphone with a built-in accelerometer and gyroscope would be suitable. The smartphone was mounted in front of the participant’s sternum, using an adjustable Action Mount chest harness (http://action-mount.com), with the accelerometer’s positive X-axis pointing upwards. A second smartphone, of the same model, was used concurrently by the researcher to start and stop recording, and to set the current trial and condition number.

The phones were running Android 7.0 and paired via Bluetooth 4.0. An application was created to record sensor data from the participant’s phone. Of primary interest was vertical acceleration, sampled at a frequency of 100 Hz. The Android API also allows for the collection of linear acceleration data (acceleration minus the effect of gravity); however, we found it to have lower precision than manually calculating the value. Therefore, we also recorded gravity sensor values, which used the built-in gyroscope, and subtracted them from the acceleration vector for the calculation of linear acceleration. For devices without a gyroscope or gravity sensor, the tilt of the device (and thus the gravity component) can be isolated using traditional techniques such as applying a low-pass filter to the acceleration signal^22^.

### Methods

After providing signed consent, participants wore a chest harness with the phone secured directly in front of their sternum. They were asked to walk, at a self-selected pace, down a 30 m hallway while performing a concurrent verbal task. The oral version of the trail making test (OTMT) was used for the low and high load conditions^23,24^. In the low load condition (OTMT-A), participants were asked to count numbers (e.g., 1, 2, 3) while walking. In the high load condition (OTMT-B), participants were asked to alternate between numbers and letters (e.g., 1, A, 2, B, 3, C) while walking. We also included a control condition where no verbal task was performed and simply involved walking from one end of the hallway to the other. Each trial type was repeated 3 times, with the load order counterbalanced between participants, giving us a total of 9 trials per participant. Participants were asked to continue walking even if they committed a counting error. Two seconds were removed from the start and end of each trial as we were interested in the period where participants were walking at their self-selected speed, rather than the periods where they were accelerating from (or decelerating to) a standing position.

Two research assistants coded the individual steps as per the procedure described in the following section. Trials were excluded from analysis if there was a lack of agreement regarding step placement between the coders, resulting in 28 excluded trials (2% of total data). Participants with over 50% excluded trials were removed from analysis entirely, resulting in the removal of two subjects.

### Calculating Gait Dynamics

**Figure 1:**
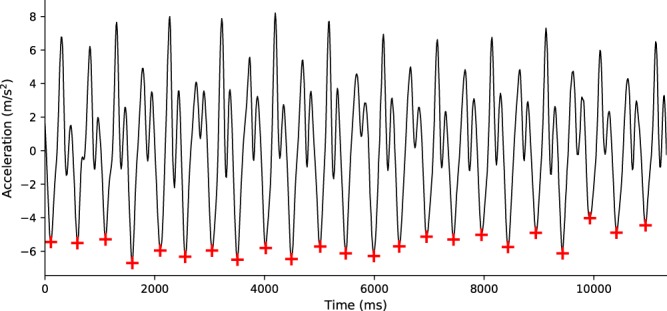
Example of typical vertical acceleration data from overground walking. Individual steps were operationalized as the valleys in the signal following an “M” shaped pattern (marked by a cross), the times between which were used to calculate the step time and step time variability.

First, linear acceleration was calculated by subtracting the gravity component from the acceleration vector. Previous work has shown that gait cycle frequency is typically below 8 Hz^25,26^, thus filtering below that frequency would remove valuable gait information. Therefore, to provide additional headroom in the filtering process, a low-pass zero-lag Butterworth filter was applied at 10 Hz to remove high-frequency noise from the linear acceleration signal. The gait cycle in vertical acceleration data typically consists of valleys occurring when the heel strikes the ground and the first peak occurring when entering the stance phase, forming a rough “M” shaped pattern in the signal^26^. The acceleration signal can be quite different for each participant due to individual differences in gait dynamics, and automated extraction of the valleys could lead to false positive identification. Research assistants blind to the hypothesis manually coded step events by identifying the valleys at the start and end of each gait cycle. A few guidelines were followed to ensure coding consistency: acceleration between steps should follow a rough “M” shaped pattern, individual differences in gait can cause deflections in the signal before or after the “M” shape, the tallest peak in the cycle usually follows the initial step of the gait cycle, and the steps are not always the lowest valleys in the cycle. See Fig. 1 for an example of typical vertical acceleration data during overground walking, where crosses in the main valleys indicate individual steps.

**Figure 2:**
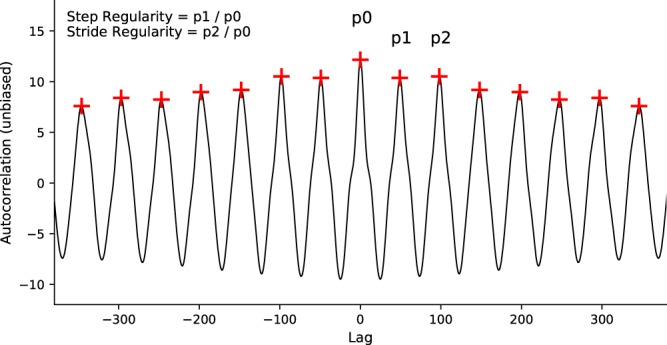
Example autocorrelation of vertical acceleration (trimmed to emphasize area around zero-lag). The signal is symmetrical around zero-lag as the time shift occurs in both directions. Correlations between a step and each subsequent/preceding step (p1, p2 etc.) are marked by crosses. Step regularity is the ratio of p1 and p0. Stride regularity is the ratio of p2 and p0.

From the times of the valleys we calculated step time (time between each step), and step time variability (standard deviation of step time). The use of acceleration data also allowed us to examine two measures of gait regularity: step regularity and stride regularity. Step regularity is the similarity in accelerative force between one step and the subsequent (contralateral) step (e.g., left foot and right foot steps) and stride regularity is the similarity between a step and the next ipsilateral step (e.g., left foot and next left foot step). These were calculated by examining the unbiased autocorrelation of the vertical acceleration signal^27^. The autocorrelation is a time-lagged correlation between a signal and itself. Given the periodic nature of gait, the time-lag allows one to examine the similarity between different steps in the gait cycle. The largest peak (p_0_) of an autocorrelation occurs at zero-lag as it is simply the correlation between a signal and itself. The next peak (p_1_) occurs when one signal is time-shifted such that the correlation will be between a step and the subsequent step. Peak p_2_ measures the correlation between a step and the next ipsilateral step (i.e. one stride). Therefore, step regularity is calculated as the ratio of p_1_ and p_0_, and stride regularity is the ratio of the p_2_ and p_0_ (Fig. 2).

### Results

We hypothesized that increasing cognitive demand would cause gait to become slower and more variable during indoor walking. Therefore, we tested whether there was a difference between the control, low, and high load conditions with regard to the previously mentioned gait parameters. The data used for the following analyses can be found in Supplementary Data S1.

#### Step Time

**Figure 3:**
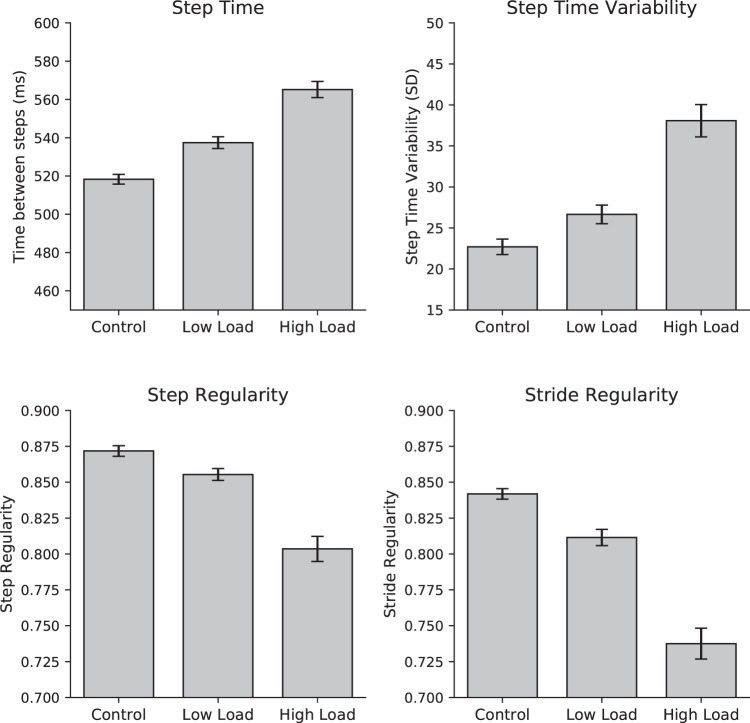
Gait parameters across different amounts of cognitive load in Experiment 1. Control = no speech; low load = Oral Trail Making Test A (OTMT-A); high load = OTMT-B. Error bars indicate standard error of the mean.

A one-way repeated measures ANOVA was conducted to determine the effect of cognitive load on step time (time between individual steps) (Fig. 3, top left). The mean (and standard deviation) of step time for the control, low, and high load conditions were 518.38 (31.28), 537.39 (37.91), and 565.20 (51.56) milliseconds, respectively. Mauchly’s test indicated a sphericity violation, χ^2^ (2) = 109.91, *p* < 0.001, and Greenhouse-Geisser adjustments were therefore used for the following tests. A significant main effect of cognitive load was observed, *F*(1.31, 197.14) = 195.49, *p* < 0.001, η_p_^2^ = 0.566. Multiple comparisons with Bonferroni adjustment indicated significant differences for all pairwise comparisons (*ps* < 0.001).

#### Step Time Variability

A one-way repeated measures ANOVA was conducted to determine the effect of cognitive load on the variability (SD) of step time (Fig. 3, top right). The mean (and standard deviation) of step time variability for the control, low, and high load conditions were 22.71 (11.62), 26.63 (14.07), and 38.08 (24.29) milliseconds, respectively. Mauchly’s test indicated a sphericity violation, χ^2^ (2) = 150.85, *p* < 0.001, and Greenhouse-Geisser adjustments were therefore used for the following tests. A significant main effect of cognitive load was observed, *F*(1.22, 183.30) = 63.58, *p* < 0.001, η_p_^2^ = 0.298. Multiple comparisons with Bonferroni adjustment indicated significant differences for all pairwise comparisons (*ps* < 0.001).

#### Step Regularity

A one-way repeated measures ANOVA was conducted to determine the effect of cognitive load on step regularity (Fig. 3, bottom left). The mean (and standard deviation) of step regularity for the control, low, and high conditions were 0.87 (0.05), 0.86 (0.05), and 0.80 (0.11), respectively. Mauchly’s test indicated a sphericity violation, χ^2^ (2) = 167.02, *p* < 0.001, and Greenhouse-Geisser adjustments were therefore used for the following tests. A significant main effect of cognitive load was observed, *F*(1.20, 179.21) = 65.19, *p* < 0.001, η_p_^2^ = 0.303. Multiple comparisons with Bonferroni adjustment indicated significant differences for all pairwise comparisons (*ps* < 0.001).

#### Stride Regularity

A one-way repeated measures ANOVA was conducted to determine the effect of cognitive load on stride regularity (Fig. 3, bottom right). The mean (and standard deviation) of stride regularity for the control, low, and high conditions were 0.84 (0.04), 0.81 (0.07), and 0.74 (0.13), respectively. Mauchly’s test indicated a sphericity violation, χ^2^ (2) = 113.94, *p* < 0.001, and Greenhouse-Geisser adjustments were therefore used for the following tests. A significant main effect of cognitive load was observed, *F*(1.30, 195.50) = 81.11, *p* < 0.001, η_p_^2^ = 0.351. Multiple comparisons with Bonferroni adjustment indicated significant differences for all pairwise comparisons (*ps* < 0.001).

### Discussion

Overall, our results show strong support for our hypothesis that cognitive load is related to gait during indoor walking in our young participant cohort. Specifically, gait became less stable under high levels of cognitive load, resulting in increases in step time and step time variability, as well as decreases in step and stride regularity. Importantly, these differences were detectable using smartphone accelerometry. Research on postural control suggests that verbal articulation may be a large contributor to dual task deficits and postural instability^28^. We included a control condition that didn’t require articulation and found that even the low load condition was enough to destabilize gait, which may provide some support for the verbal articulation hypothesis. However, our results also show significant differences between our low and high load conditions (both of which contain a verbal component), across multiple gait parameters, suggesting that cognitive load may act as an additional contributor to young adult gait control, over and above any effect verbal articulation may have.

## Experiment 2

While it has traditionally been challenging to measure gait outdoors, it is also difficult to quantify the cognitive demand of an outdoor environment. Therefore, before we can study the relationship between environmentally induced cognitive load and gait in outdoor environments, we first need an operationalization of load. One operational definition of cognitive demand is the fractal dimension (FD) of a visual scene. High-fractal images contain repetitive information, and their repetitive nature results in increased perceptual fluency and confers lower cognitive demand than low-fractal counterparts^29^. Furthermore, fractals are often found in natural environments^30^ and it is therefore not surprising that images of nature are found to have a restorative effect on attention^11^. While there is some evidence to support the relationship between FD and cognitive load in computer-generated imagery^29^, we ran a validation study to test whether FD of real outdoor photographs was related to cognitive load. The primary objective was to determine whether 1) fractal dimension can distinguish between different types of outdoor environments, and 2) outdoor images with low fractal dimension are more cognitively demanding to process than high-fractal images. To test this, we showed participants images of urban and natural scenes and timed their responses to a question about the image. Fractal dimension was then calculated for each image and correlated with their response time. The goal was to validate the relationship between FD and cognitive load, with the idea of using FD as a measure of cognitive demand in outdoor walking experiments.

### Participants

A power analysis was conducted to determine minimum sample size for this study. An effect size of 0.3 was used, which was estimated from a study examining the relationship between fractal dimension and cognitive function^29^. This effect size is also in line with the smallest effect observed in Experiment 1. The power analysis showed that a minimum of 37 participants would be required to achieve 80% power. A total of 52 undergraduate student volunteers (mean age = 20.86, *SD* = 2.33, 9 male) were recruited from the Greater Vancouver area and received course credit for their time.

### Methods

**Figure 4:**
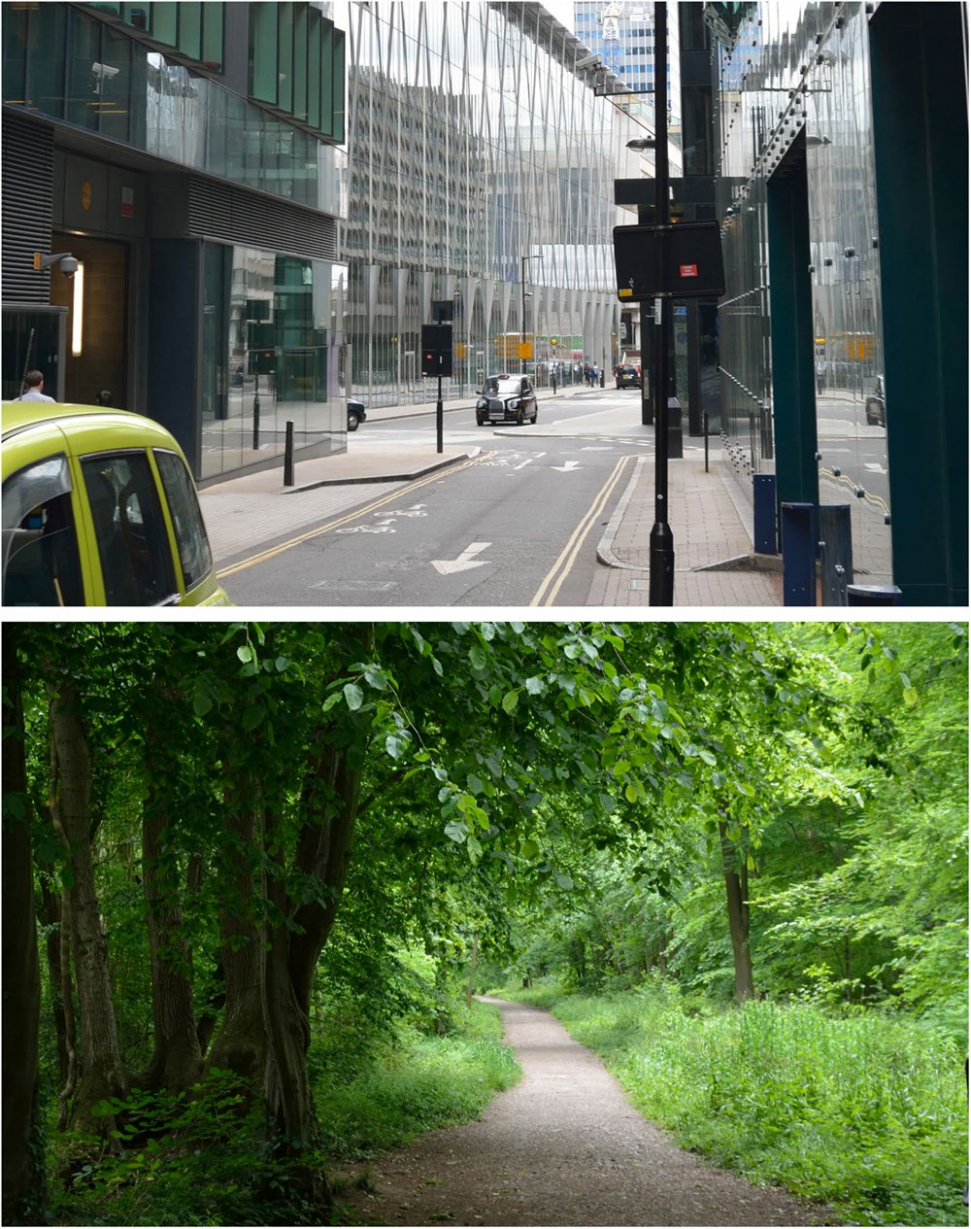
Example of urban (top) and nature (bottom) photographs presented to participants in Experiment 2.

Participants saw 50 photos of urban scenes and 50 photos of natural scenes in randomized order (Fig. 4). Each image had pixel dimensions of 1280 × 800, was shown for 5 seconds, and presentation order was randomized between participants. Upon seeing each image, participants were asked “how uncomfortable is this image to view?”, which was rated using a 7-point Likert scale. This question was asked primarily to focus the participant’s attention on the image. Their image rating was recorded along with their response time, and the Minkowski–Bouligand fractal dimension was calculated for each image^31^. The colour images were normalized, converted to grayscale, and finally binarized using the mean image value, before the box counting algorithm was run over a range of box sizes to calculate fractal dimension.

### Results

We predicted that 1) nature scenes would have a higher fractal dimension than urban scenes, and more importantly 2) participants would respond faster to images with higher fractal dimension owing to their reduced cognitive demand. The data used for the following analyses can be found in Supplementary Data S2. First, we conducted Welch’s t-test to determine whether there was a difference in FD between urban and nature images. The mean (and standard deviation) of FD for the urban and nature images were 1.62 (0.09) and 1.74 (0.09), respectively. A significant difference between the image types was observed, *t*(97.84) = 6.97, *p* < 0.001. This result suggests that images of nature have a higher fractal dimension than urban images.

Next, we employed a linear mixed-effects model to determine the relationship between FD, self-reported visual discomfort, and response time. All variables were first standardized, then we predicted response time from FD and visual discomfort, modelling random intercepts and random slopes for both relationships. The random effects were grouped by subject as we expected the relationships to be clustered. FD was found to be significantly negatively related to response time, *β* = −0.12, *t*(51.34) = −7.56, *p* < 0.001. This finding suggests that as fractal dimension increases, response time to the image decreases (i.e. becomes faster). Additionally, self-reported visual discomfort was positively related to response time, *β* = 0.18, *t*(48.12) = 6.16, *p* < 0.001, showing that participants responded slower to images they found more uncomfortable.

### Discussion

Outdoor environments naturally impose demands on cognition; for example, urban environments have high attentional demands while images of nature are thought to have a restorative effect on attention^11^. Additionally, research suggests that high-fractal imagery imposes lower cognitive demands, thus freeing resources for concurrent tasks^29^. Our results are in line with these findings, showing that 1) images of nature have a higher fractal dimension than urban images, and 2) high-fractal images are associated with faster responses. That high-fractal dimension was associated with faster responses suggests that high-fractal scenes may be easier to process and less cognitively demanding. Furthermore, FD was predictive of response time independent of any contribution from self-reported visual discomfort. It is possible that participants simply preferred nature scenes, resulting in faster responses, and our results cannot exclude this possibility as we did not measure image preference. However, research using computer-generated fractal imagery shows that fractal dimension is related to cognitive function^29^, and this is independent of factors such as preference for nature scenes (as only computer-generated shapes were used). Overall, these findings present fractal dimension as a good candidate for quantifying environmentally induced cognitive load in outdoor walking environments.

## Experiment 3

To assess the relationship between gait dynamics and cognitive load during outdoor walking we used a smartphone to record vertical acceleration during a walk along a pre-chosen outdoor route. Cognitive load along the route was operationalized as fractal dimension (FD) of photos captured at a series of locations along the walk. The results from Experiment 2, and from studies using computer-generated fractal images^29^, show that high fractal dimension confers low cognitive demand. We therefore used the phone’s camera to take photographs at preselected points along the walking route, calculated fractal dimension for each photograph, and correlated it with gait parameters from the same time point as the image.

### Participants

To determine minimum sample size, we conducted a Monte Carlo simulation for a mixed-effects model using the simR software package^32^. Results from Experiment 1 showed the smallest effect size for the relationship between cognitive load and gait to be <0.30; however, walking outdoors introduces a lot of noise due to the uncontrolled environment (e.g. other pedestrians, variability in visual stimuli, weather conditions). Therefore, to avoid being underpowered, we used a conservative effect size estimate of 0.05 in case outdoor effect sizes are considerably smaller than those found in controlled settings. The simulation indicated that 228 individual data points would be needed to achieve 80% power, which, for our study design, amounts to a minimum of 29 subjects. Participants were recruited from the University of British Columbia and received monetary compensation for their time. Participants were excluded if they were over 60 years of age as cognitive function declines with age and will likely confound any observed relationships^33^. Furthermore, some participants had an incorrectly positioned smartphone, which would negatively impact the calculation of fractal dimension (described in more detail below). Our final sample consisted of 38 participants (mean age = 26.04, *SD* = 6.83, 13 male).

### Methods

**Figure 5:**
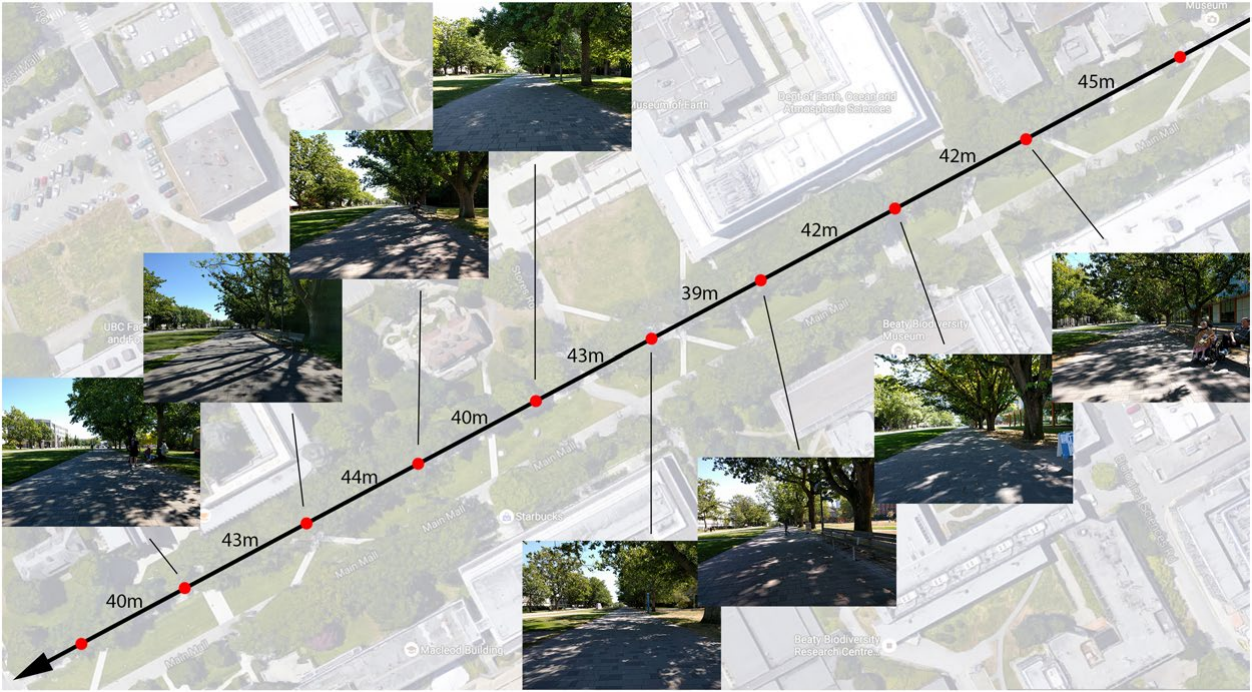
Checkpoint photos from Experiment 3 for a randomly-selected participant. Map data: Google.

After providing signed consent, participants wore a chest harness with a smartphone phone secured directly in front of their sternum, and their height was measured. A research assistant used a second smartphone, connected via Bluetooth, to instruct the *participant’s* phone to take photographs at various points along the walking route. This provided photos of the walking environment from the perspective of the participant at the time of the actual walk. A 520 m walking route was used for the study. The route was selected due to having variance in the visual scene, few physical obstructions, and consistency in the walking surface (concrete, with no major bumps or changes in inclination). Participants were only tested on days with no rain to ensure that gait dynamics were not affected by a change in the conditions of the walking surface. Ten approximately equidistant checkpoints were selected along the route. The first checkpoint was excluded from analysis as the participant was accelerating up to their normal walking speed. Similarly, the final checkpoint was excluded due to deceleration to standing at the end of the route. Ultimately, only the middle eight checkpoints were analyzed (Fig. 5).

Participants started at the first checkpoint and were asked to walk at a self-selected speed to the end of the route at the final checkpoint. They were instructed to look directly ahead while walking, not to interact with other pedestrians, and to maintain as straight a path as possible while avoiding oncoming traffic (e.g. bicycles, pedestrians). Participants were told that the research assistant would follow them at a distance of 5 m. The research assistant used their smartphone to take a photograph from the participant’s phone at each of the checkpoints.

After data collection, the Minkowski–Bouligand fractal dimension was calculated using the box-counting technique for each photograph^31^. Gait dynamics were calculated from the 10-seconds following each timestamped photo. For correct calculation of fractal dimension, the participant’s phone must be positioned correctly on the harness. Participants were excluded if the environment’s horizon line was not within the middle third of the image. Additionally, individual checkpoints were excluded if the participant deviated from the path (e.g. walked on grass), or if the photo was accidentally taken too early. These exclusions were already considered in our sample description in the previous section. Finally, we coded the number of other pedestrians on the walking path as an additional source of cognitive load, which was used as a covariate in the following statistical models.

### Results

We hypothesized that fractal dimension (FD) of an image would be associated with the dynamics of gait. Specifically, as fractal dimension increases (and thus cognitive load decreases), step time and step time variability would decrease, while step and stride regularity would increase. These predictions stem from our findings in Experiment 1. The data used for the following analyses can be found in Supplementary Data S3.

We employed linear mixed-effects models to formally test for the presence of each predicted relationship. All variables were first standardized at the raw score level using the grand mean. In predicting each of the gait parameters, we began by including participant height and number of other pedestrians (counted from the photos) as covariates, as they would likely impact gait dynamics. Next, we disaggregated fractal dimension into two components to disentangle between and within-subject fluctuations in FD^34,35^, both of which were used as separate predictors in the model. This was accomplished by calculating mean FD for each participant from their set of eight photos, which captures between-subject fluctuations in FD that may arise from changes in environmental conditions such as weather and differences in lighting/shadows. Next, for our primary predictor of interest, we calculated each photo’s deviation from that individual’s previously calculated mean FD, which captures the within-subject changes in fractal dimension for that individual. In sum, each gait parameter was predicted by height, number of pedestrians, mean FD, and deviations in FD. Random effect components were added for both the intercept and slope of the FD deviation values, clustered by subject. For the following models, parameters were estimated using restricted maximum likelihood; Satterthwaite approximations were used for the degrees of freedom, and confidence intervals were estimated using percentile bootstrap.

#### Step Time

For the random effects, the standard deviation around the mean intercept was found to be significant, *SD* = 0.87, 95% CI [0.66, 1.09], suggesting a significant degree of variation in step time between subjects. Additionally, the standard deviation around the FD deviation slope values was significant, *SD* = 0.004, 95% CI [0.001, 0.06], suggesting that the relationship between FD and step time is different for each subject. For the fixed effects, height was significantly positively associated with step time, *β* = 0.64, 95% CI [0.35, 0.94], *t*(34.88) = 4.31, *p* < 0.001. However, none of the other predictors showed a significant relationship with step time (pedestrian count: *β* = −0.003, 95% CI [−0.03, 0.03], *t*(261.40) = −0.18, *p* = 0.86. FD mean: *β* = 0.35, 95% CI [−0.36, 1.13], *t*(34.94) = 0.92, *p* = 0.37. FD deviation: *β* = −0.004, 95% CI [−0.03, 0.03], *t*(246.80) = −0.31, *p* = 0.76).

#### Step Time Variability

For the random effects, the standard deviation around the mean intercept was found to be significant, *SD* = 0.97, 95% CI [0.73, 1.20], suggesting a significant degree of variation in step time variability between subjects. Additionally, the standard deviation around the FD deviation slope values was significant, *SD* = 0.02, 95% CI [0.002, 0.16], suggesting that the relationship between FD and step time variability is different for each subject. For the fixed effects, none of the predictors were significantly associated with step time variability (height: *β* = 0.10, 95% CI [−0.23, 0.43], *t*(34.90) = 0.60, *p* = 0.55. pedestrian count: *β* = 0.03, 95% CI [−0.04, 0.10], *t*(266.30) = 0.78, *p* = 0.44. FD mean: *β* = −0.32, 95% CI [−1.20, 0.45], *t*(35.13) = −0.73, *p* = 0.47. FD deviation: *β* = −0.004, 95% CI [−0.07, 0.07], *t*(224.43) = −0.10, *p* = 0.92).

#### Step Regularity

For the random effects, the standard deviation around the mean intercept was found to be significant, *SD* = 1.19, 95% CI [0.91, 1.48], suggesting a significant degree of variation in step regularity between subjects. Additionally, the standard deviation around the FD deviation slope values was significant, *SD* = 0.04, 95% CI [0.006, 0.12], suggesting that the relationship between FD and step regularity is different for each subject. For the fixed effects, none of the predictors were significantly associated with step regularity (height: *β* = −0.02, 95% CI [−0.38, 0.40], *t*(34.75) = −0.10, *p* = 0.92. pedestrian count: *β* = −0.01, 95% CI [−0.07, 0.04], *t*(262.57) = −0.51, *p* = 0.61. FD mean: *β* = −0.25, 95% CI [−1.37, 0.79], *t*(35.12) = −0.49, *p* = 0.63. FD deviation: *β* = 0.02, 95% CI [−0.03, 0.07], *t*(136) = 0.83, *p* = 0.41).

#### Stride Regularity

For the random effects, the standard deviation around the mean intercept was found to be significant, *SD* = 0.66, 95% CI [0.47, 0.85], suggesting a significant degree of variation in stride regularity between subjects. Additionally, the standard deviation around the FD deviation slope values was significant, *SD* = 0.08, 95% CI [0.01, 0.23], suggesting that the relationship between FD and stride regularity is different for each subject. For the fixed effects, none of the predictors were significantly associated with stride regularity (height: *β* = −0.01, 95% CI [−0.22, 0.22], *t*(33.44) = −0.06, *p* = 0.95. pedestrian count: *β* = −0.04, 95% CI [−0.13, 0.06], *t*(281.38) = −0.75, *p* = 0.46. FD mean: *β* = −0.30, 95% CI [−0.94, 0.24], *t*(34.40) = −1.03, *p* = 0.31. FD deviation: *β* = 0.07, 95% CI [−0.02, 0.17], *t*(96.93) = 1.37, *p* = 0.18).

### Discussion

Our results failed to find support for our hypothesis that fractal dimension of outdoor photos is related to gait dynamics. The significant random effects (intercept and slope) across the models are not surprising as we would expect gait to vary between individuals. Additionally, the only significant fixed effect predictor was for height in the relationship between FD and step time. Specifically, we found that as participant height increased, their time between steps also increased. This is expected as taller individuals are likely to have a longer stride length, thus longer time between each step.

One possible reason for the null results is a lack of sufficient variance in fractal dimension across the checkpoints. Figure 5 shows that the visual scene along the route is quite similar, with all images containing high amounts of foliage, constant shadowing on the ground, and little to distinguish one checkpoint from another. In Experiment 1 we showed that explicit manipulation of cognitive load can lead to changes in gait. However, when load is inherent to the environment the effect may not be as strong, and thus larger variability in load may be required to see an effect in a correlational real-world design.

## Experiment 4

To test whether our null results from Experiment 3 may have been due to low variability in our predictors, a new walking route was chosen that contained more variability in fractal dimension across checkpoints.

### Participants

Participants were again recruited from the University of British Columbia and received monetary compensation for their time. Participants were excluded for the following reasons: hardware issues (data saving problems due to Bluetooth disconnections), one participant was constantly adjusting the harness which led to a lot of noise in the signal, and incorrectly positioned phone (as described in the previous experiment). Our final sample consisted of 38 participants (mean age = 24.79, *SD* = 6.04, 15 male).

### Methods

**Figure 6:**
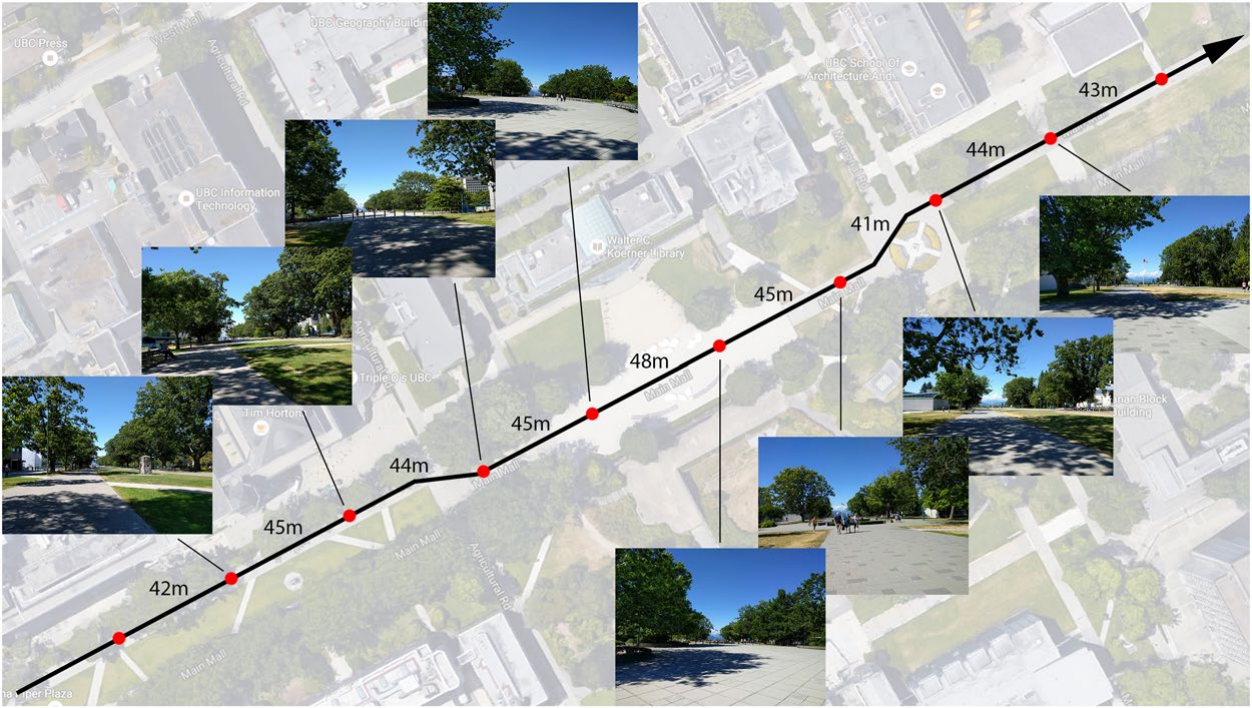
Checkpoint photos from Experiment 4 for a randomly-selected participant. Map data: Google.

The procedure was identical to Experiment 3, with the only difference being a new walking route. During location scouting for the new route, a research assistant took 11 photos (on different days) at each checkpoint of the previous route used in Experiment 3. Variability in fractal dimension was then calculated and used as a baseline value for comparison against candidate routes. This process was repeated for each new candidate route. The chosen route (Fig. 6) was approximately 480 m in length and was selected for increased variability in the visual scene at each checkpoint (e.g., differences in amount of foliage, ground shadows, visible sky, larger variability in fractal dimension induced by higher amounts of built environment), while still maintaining consistency in the walking surface (concrete, with no major bumps or changes in inclination). The data used for the following analyses can be found in Supplementary Data S4.

### Results

#### Fractal Dimension Variability

**Figure 7:**
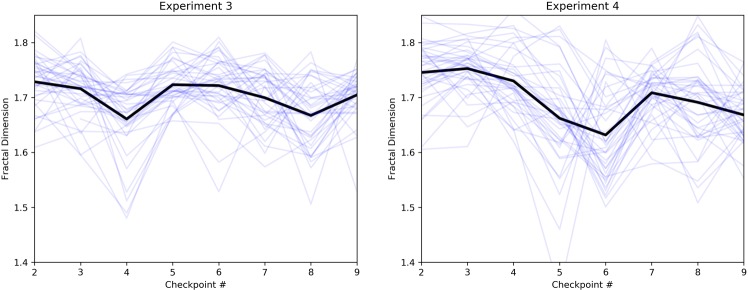
Mean fractal dimension and individual values for all participants across all checkpoints in both outdoor experiments. The traces suggest there is more variability in fractal dimension in Experiment 4.

To show that the new route has more variability in fractal dimension compared to Experiment 3, we graphed the mean FD value for each checkpoint, along with the trace for each individual subject (Fig. 7). The graphs show that the new route has a wider range of FD values, as well as a larger amount of variance, across the checkpoints. This was confirmed using Levene’s test for homogeneity of variance between the two experiments. The test shows a significant difference in variance of FD between Experiments 3 and 4, *F*(1, 599) = 23.29, *p* < 0.001. The difference in mean FD between the two experiments was not found to be significant, *t*(554.3) = 0.68, *p* = 0.50, suggesting that average load between the two experiments was similar.

#### Step Time

For the random effects, the standard deviation around the mean intercept was found to be significant, *SD* = 0.78, 95% CI [0.60, 0.98], suggesting a significant degree of variation in step time between subjects. The standard deviation around the FD deviation slope values was also significant, *SD* = 0.02, 95% CI [0.002, 0.06], suggesting that the relationship between FD and step time is different for each subject. For the fixed effects, height was significantly positively associated with step time, *β* = 0.49, 95% CI [0.24, 0.74], *t*(35.13) = 3.91, *p* < 0.001. Importantly, FD deviation was significantly negatively associated with step time, *β* = −0.04, 95% CI [−0.06, −0.02], *t*(25.53) = −3.77, *p* < 0.001, suggesting that time between steps becomes shorter as fractal dimension increases (i.e. cognitive load decreases). However, none of the other predictors showed a significant relationship with step time (pedestrian count: *β* = −0.01, 95% CI [−0.04, 0.01], *t*(259.84) = −1.14, *p* = 0.26. FD mean: *β* = 0.01, 95% CI [−0.62, 0.56], *t*(34.48) = 0.05, *p* = 0.96).

**Figure 8:**
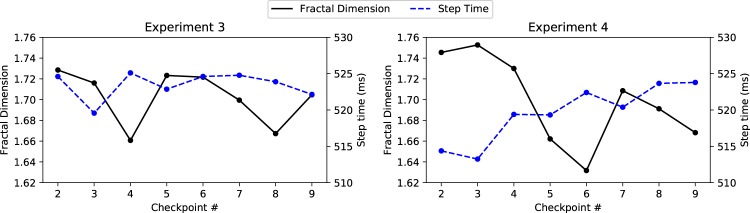
Mean fractal dimension and step time across all checkpoints in both outdoor experiments. Experiment 3 shows a narrower range of parameter values, suggesting reduced variability across the walk. Furthermore, Experiment 4 shows an inverse relationship between FD and step time, where step time increases once FD starts to decline. The other gait parameters are not plotted as they were not significantly related to FD.

The significant effect of step time differs from the finding in Experiment 3, where no such relationship was observed. The differences between Experiments 3 and 4 can be further highlighted by plotting mean FD and step time across all checkpoints for each experiment (Fig. 8). The plots show two notable data patterns. First, the FD and step time values in Experiment 3 span a narrower range compared to Experiment 4, suggesting, again, less variability in parameter values across the walk. Second, Experiment 4 shows a clear inverse relationship between FD and step time: low step time values are seen when FD is high (e.g. checkpoints 2 to 3), and when FD begins to decline we see a rise in step time (e.g. checkpoints 3 to 6). This relationship is not as pronounced in Experiment 3, likely due to reduced variability in parameter values.

#### Step Time Variability

For the random effects, the standard deviation around the mean intercept was found to be significant, *SD* = 0.83, 95% CI [0.65, 1.04], suggesting a significant degree of variation in step time variability between subjects. Additionally, the standard deviation around the FD deviation slope values was significant, *SD* = 0.10, 95% CI [0.03, 0.16], suggesting that the relationship between FD and step time variability is different for each subject. For the fixed effects, none of the predictors were significantly associated with step time variability (height: *β* = 0.10, 95% CI [−0.17, 0.32], *t*(36.73) = 0.79, *p* = 0.44. pedestrian count: *β* = 0.01, 95% CI [−0.04, 0.05], *t*(260.77) = 0.26, *p* = 0.80. FD mean: *β* = 0.28, 95% CI [−0.25, 0.80], *t*(34.12) = 0.98, *p* = 0.33. FD deviation: *β* = −0.02, 95% CI [−0.07, 0.03], *t*(38.57) = −0.70, *p* = 0.49).

#### Step Regularity

For the random effects, the standard deviation around the mean intercept was found to be significant, *SD* = 0.75, 95% CI [0.57, 0.94], suggesting a significant degree of variation in step regularity between subjects. Additionally, the standard deviation around the FD deviation slope values was significant, *SD* = 0.01, 95% CI [0.002, 0.08], suggesting that the relationship between FD and step regularity is different for each subject. For the fixed effects, none of the predictors were significantly associated with step regularity (height: *β* = −0.04, 95% CI [−0.27, 0.22], *t*(35.09) = −0.32, *p* = 0.75. pedestrian count: *β* = −0.01, 95% CI [−0.05, 0.03], *t*(266.39) = −0.50, *p* = 0.62. FD mean: *β* = 0.13, 95% CI [−0.41, 0.68], *t*(34.83) = 0.47, *p* = 0.64. FD deviation: *β* = 0.02, 95% CI [−0.02, 0.06], *t*(215.37) = 0.98, *p* = 0.33).

#### Stride Regularity

For the random effects, the standard deviation around the mean intercept was found to be significant, *SD* = 0.47, 95% CI [0.33, 0.62], suggesting a significant degree of variation in stride regularity between subjects. Additionally, the standard deviation around the FD deviation slope values was significant, *SD* = 0.01, 95% CI [0.002, 0.20], suggesting that the relationship between FD and stride regularity is different for each subject. For the fixed effects, none of the predictors were significantly associated with stride regularity (height: *β* = 0.08, 95% CI [−0.08, 0.27], *t*(34.98) = 0.93, *p* = 0.36. pedestrian count: *β* = −0.04, 95% CI [−0.13, 0.07], *t*(295.63) = −0.70, *p* = 0.49. FD mean: *β* = 0.09, 95% CI [−0.30, 0.48], *t*(35.44) = 0.43, *p* = 0.67. FD deviation: *β* = 0.02, 95% CI [−0.07, 0.11], *t*(260.86) = 0.52, *p* = 0.60).

### Discussion

We hypothesized that the null results in Experiment 3 were due to low variability in the fractal dimension measure across participants and checkpoints. After increasing FD variability in the present experiment, our results showed that step time became shorter when FD increased (i.e. cognitive load decreased). This supports the step time finding in Experiment 1; however, the other gait parameters were still not related to fractal dimension. The overall data pattern will be discussed in more detail in the general discussion section.

## General Discussion

Research has demonstrated that an increase in cognitive load can result in increased gait variability and slower overall walking speed^13^. The external environment also imposes load on our cognitive systems; however, most gait research has been conducted in a laboratory setting and little work has demonstrated how load imposed by real-world environments impacts gait dynamics during outdoor walking. Overall, our results suggest that, during indoor walking, task-induced increased cognitive load impacted a range of gait parameters such as step time and step time variability. The impact of environmental cognitive load on gait, however, was not as pronounced, with increased load associated only with step time increase during outdoor walking. This suggests that the intensity of experienced load moderates the degree to which gait dynamics are affected. Overall, the present work shows that cognitive load is related to young adult gait during both indoor and outdoor walking, and importantly, that smartphones can be used as gait assessment tools in environments where gait dynamics have traditionally been difficult to measure. Given our findings, several theoretical questions and issues follow.

Cognitive load induced by everyday environments is, potentially, of lower intensity than load imposed by an explicit dual-task, and it is possible that changes in cognitive demand induced by everyday man-made outdoor environments are too low to bring about changes in all measured gait parameters. Our results show that, compared to task-related cognitive load changes in indoor walking, the impact of environmental cognitive load on outdoor gait is less pronounced, with only step time being affected by increased environmental load. The low load levels in outdoor environments, coupled with generally better cognitive function in young adults relative to older adults^33,36^, could result in young adults only needing to modulate step time to compensate for changes in environmental load. Future studies can test this hypothesis by exposing individuals to a dual-task while walking outdoors and seeing whether fractal dimension impacts gait after baseline levels of experienced load have increased or testing individuals in more cognitively demanding environments such as urban city centers or street intersections. Additionally, a body of works shows that increased cognitive load can result in decreased gait speed and increased gait variability in older adults^37–39^. However, like with young adults, most of the older adult gait literature is conducted indoors and it is currently unknown how gait is affected by environmental load during outdoor walking in that population. We predict that lower levels of environmental load are needed to see changes in older adult gait dynamics, but this remains an empirical question and is another potential avenue for future research.

While fractal dimension has been linked to cognitive load and perceptual fluency^29^, and the relationship is supported by our findings in Experiment 2, it remains unclear whether a photo from chest level is a good representation of where participants were actually looking while walking through the environment. Although we instructed participants to look directly ahead during their walk, we can’t guarantee that this instruction was followed for the entire route, and any head turns or changes in gaze location may result in reduced validity of our fractal dimension measure. Some work has shown that gaze is typically directed towards the ground while walking outdoors over rough terrain, but is less focused on the ground plane when walking over flat terrain^6^. As both Experiments 3 and 4 contained flat ground walking, it may be prudent for future studies to utilize head-mounted cameras or eye-trackers for more precise localization of gaze during the walk.

Traditional methods of gait assessment can be cost-prohibitive, and the present study demonstrates the use of smartphone-based techniques for this task, confirming that smartphones can be used as relatively cheap, accessible alternatives for examining the relationship between gait and cognition in outdoor environments. While some have studied outdoor gait using state-of-the-art mobile motion capture systems^6^, smartphones offer a more robust methodology that is both relatively inexpensive and more readily available. Modern smartphones have built-in accelerometers and accelerometry is commonly used in the study of gait and postural balance, which has been shown to have accuracy similar to force platform-based techniques^40^. Analysis of gait patterns from accelerometers can provide the ability to differentiate between habitual fallers and healthy adults^41^, and can be used as a diagnostic tool for rheumatoid arthritis^42^. Although it should be noted that data accuracy and consistency depends on placement of the accelerometer^43–45^. Smartphones have only been used to study gait in a small handful of studies^42^; however, smartphone-based methods could allow researchers to ask new research questions, and have some additional benefits over traditional methods. For example, while they can be used to examine gait in natural, outdoor environments, they can also be employed for monitoring gait in home environments owing to their portability. Additionally, smartphones can be used as portable testing computers, allowing for remote administration of questionnaires or cognitive tasks, which can then be correlated with gait measures during the same time-period. This approach could be extended using Ecological Momentary Assessment, which would allow for direct examination of within-person variability in gait dynamics and cognitive function^46,47^. Finally, smartphone applications have the potential for wide deployment on application stores (e.g., Apple App Store, Google Play Store), allowing for 1) faster acquisition of data, and 2) access to a larger, more diverse population; thus, allowing for cross-cultural comparison studies. There are some drawbacks compared to traditional methods (e.g. 3D motion capture), however, as distance-based metrics such as stride length are difficult to obtain from acceleration data. While some techniques exist to estimate distance metrics^48,49^, the direct transformation of acceleration requires double numerical integration of the signal, which may be problematic given the noisy nature of acceleration data. As such, the validity and accuracy of distance-based gait measures derived from acceleration data through integration techniques remains an empirical question.

In conclusion, our results suggest that in young adults both dual task induced as well as sensory environment induced cognitive load are related to changes in gait dynamics, although the effect was attenuated in the latter, possibly owing to reduced levels of cognitive load changes when walking outdoors. Furthermore, our findings show that smartphone-based accelerometry is sensitive enough to detect these relationships, thus allowing for future gait studies to be conducted in more natural environments where gait dynamics have traditionally been difficult to measure.

## Supplementary information

Supplementary Data S1

Supplementary Data S2

Supplementary Data S3

Supplementary Data S4

## Data Availability

All data generated or analysed during this study are included in this published article (and its Supplementary Information files).
